# Survey of pharmacists on their roles and perceptions of outpatient parenteral antimicrobial therapy in the United States

**DOI:** 10.1017/ash.2022.40

**Published:** 2022-04-22

**Authors:** Christina G. Rivera, Kristin C. Mara, Monica V. Mahoney, Keenan L. Ryan

**Affiliations:** 1 Mayo Clinic Department of Pharmacy, Rochester, Minnesota; 2 Mayo Clinic Department of Quantitative Health Sciences, Rochester, Minnesota; 3 Beth Israel Deaconess Medical Center, Boston, Massachusetts; 4 University of New Mexico Hospital, Albuquerque, New Mexico

## Abstract

**Objectives:**

To define outpatient parenteral antimicrobial therapy (OPAT) clinical pharmacy practice across the United States, specifically pharmacist functions, design of pharmacist involvement, and to compare pharmacist training of those who practice in OPAT to infectious diseases pharmacists who do not.

**Methods:**

A survey of a possible 32 questions was emailed to the American College of Clinical Pharmacists (ACCP) Infectious Diseases Practice and Research Network (PRN) e-mail list. Results were focused on US-based respondents.

**Participants:**

In total, 87 pharmacists responded; 27 of these pharmacists (31%) practiced in OPAT.

**Results:**

Training background did not differ between groups. Programs with an OPAT pharmacist were more likely to have a formal OPAT team compared to those without an OPAT pharmacist (*P* < .001). OPAT pharmacists were early in their careers with 66.7% practicing <5 years in OPAT. Most OPAT pharmacists (66.7%) practiced at an academic medical center with a median full-time equivalent (FTE) of 0.6. Moreover, 63% utilized a collaborative practice agreement and 81.5% shared job functions with other pharmacist roles, most commonly antimicrobial stewardship. Few OPAT programs involved a dispensing component (28%). The median daily census was 43 patients followed by an OPAT pharmacist. Pharmacists performed a variety of tasks in OPAT.

**Conclusion:**

Pharmacist nondispensing involvement in OPAT is an emerging trend in the United States with wide variability in program structure and pharmacist tasks. A ratio of 1 OPAT pharmacist for every 45–70 OPAT patients is proposed to facilitate expansion of pharmacist clinical practice in OPAT.

Outpatient parenteral antimicrobial therapy (OPAT) is defined as the administration of intravenous antimicrobials, outside the acute care setting, on 2 separate calendar days.^1^ As the US OPAT registry ceased to exist in 2000, no national databases in the United States currently estimate OPAT services, patients, or clinicians. What information is available is piecemeal from several reports. Oral antimicrobials for complex infections, or complex outpatient antimicrobial therapy (COpAT), is emerging as a component incorporated into many OPAT programs.

In 1998, it was estimated that >250,000 patients received OPAT in the United States annually.^2^ With changes in medical reimbursement structures, patient preferences, and global pandemics, it is reasonable to presume this number has since increased. In a 2014 survey of infectious diseases (ID) physicians, 118 indicated they had dedicated OPAT teams at their institutions, but it is unknown how many unique programs this represents.^3^ There is a dearth of information regarding the role of pharmacists in OPAT because most practice surveys have been sent only to ID physicians.^4^ Likewise, only 33 postgraduate year 2 (PGY2) ID pharmacy residency programs indicated offering an OPAT rotation, implying some sort of program or preceptor availability.^5^ This represented less than half the programs surveyed. While implied, it is still unknown how many of these represent unique programs.

OPAT programs are expanding, as are the opportunities for clinical ID pharmacist involvement. The current state of clinical (nondispensing) roles and functions being performed by pharmacists in OPAT is unknown. Therefore, we sought to quantify how many pharmacists within the United States have formal roles within OPAT. Additionally, we sought to determine the clinical functions performed by pharmacists and the design of pharmacist involved in OPAT clinics. We also compared the training of pharmacists who practice in OPAT to ID pharmacists who do not.

## Methods

A survey utilizing Research Electronic Data Capture (REDCap) created by the authors (Supplement 1), underwent review by 3 independent clinical pharmacist researchers and was approved by the pharmacy research committee (Minnesota site). Information was limited to only pharmacists practicing in the United States. Branching logic was used to differentiate between pharmacists with and pharmacists without OPAT clinic involvement. Pharmacists without OPAT involvement were asked 10 demographic questions and 1 question surrounding the composition of OPAT teams, or lack thereof. Pharmacists with OPAT clinics were asked 11 demographic questions and 21 OPAT-specific questions. Responses for each question were optional and anonymous.

An e-mail invitation to participate included the REDCap survey link and was sent to the American College of Clinical Pharmacy (ACCP) ID Practice and Research Network (PRN) listserv March 1, 2021. The survey remained open until March 31, 2021. In addition, 2 e-mail reminders were sent. The ID PRN is a network of clinical pharmacists with an interest in ID with ∼2,000 members in March 2021. To increase participation, 5 cash cards were offered as remuneration. Participation in the raffle was uncoupled from the survey completion.

This study received institutional review board approval from all 3 institutions.

## Results

In total, 87 pharmacists responded to the survey; 27 of these (31%) indicated an OPAT practice (Table 1). Most respondents held a Doctor of Pharmacy degree (97.7%), which was reflected by years of pharmacist license: 33.3% had been licensed <5 years, 31.0% had been licensed 5–10 years, and 13.8% had been licensed 10–15 years. Most had additional postgraduate training: 58.6% had completed a PGY1, 62.1% had completed an ID PGY2, 26.4% had completed antimicrobial stewardship certificate training, and others had additional training. Only 4 respondents (1 in 4 in OPAT practice) did not have additional training beyond their pharmacist degree. Respondents represented 31 distinct states. Academic medical centers were the most frequent site of employment (49.4%).

**Table 1.** Pharmacist Demographics

Characteristic	Total (n = 87)	No OPAT Practice (n = 60)	OPAT Practice (n = 27)	*P* Value
Doctor of pharmacy degree	85 (97.7)	58 (96.7)	27 (100.0)	.66
**Postgraduate training**				
PGY1	51 (58.6)	34 (56.7)	17 (63.0)	.58
PGY2-Infectious Diseases	54 (62.1)	35 (58.3)	19 (70.4)	.28
ID Fellowship	5 (5.7)	4 (6.7)	1 (3.7)	.58
Antimicrobial stewardship certificate	23 (26.4)	18 (30.0)	5 (18.5)	.26
None	4 (4.6)	3 (5.0%)	1 (3.7%)	.79
**Years as a license pharmacist**				.84
<5 y	29 (33.3)	21 (35.0)	8 (29.6)	
5–10 y	27 (31.0)	18 (30.0)	9 (33.3)	
10–15 y	12 (13.8)	7 (11.7)	5 (18.5)	
>15 y	19 (21.8)	14 (23.3)	5 (18.5)	
**Years practicing in ID**				.95
<5 y	43 (50.0)	30 (50.8)	13 (48.1)	
5–10 y	25 (29.1)	17 (28.8)	8 (29.6)	
10–15 y	9 (10.5)	4 (6.8)	5 (18.5)	
>15 y	9 (10.5)	8 (13.6)	1 (3.7)	
Missing	1	1	0	
**Years practicing in OPAT**				
<5 y			18 (66.7)	
5–10 y			5 (18.5)	
10–15 y			2 (7.4)	
>15 y			2 (7.4)	
**Practice setting**				.092
Academic medical center/hospital	43 (49.4)	25 (41.7)	18 (66.7)	
Community hospital	30 (34.5)	26 (43.3)	4 (14.8)	
VA/Government hospital	5 (5.7)	3 (5.0)	2 (7.4)	
Infusion clinic, hospital owned	3 (3.4)	1 (1.7)	2 (7.4)	
Infusion clinic, privately owned or for profit	1 (1.1)	1 (1.7)	0	
Other	5 (5.7)	4 (6.7)	1 (3.7)	

Note. PGY1 post-graduate year 1; PGY2 post-graduate year 2; ID infectious diseases; OPAT outpatient parenteral antimicrobial therapy.

Although there were no statistical differences in demographics between pharmacists with and without OPAT practices, those with OPAT practices tended to have higher rates of PGY2-ID training (70.4% vs 58.3%, respectively). Additionally, employment within a community hospital corresponded with lower reports of OPAT pharmacist practice.

**Fig. 1.**
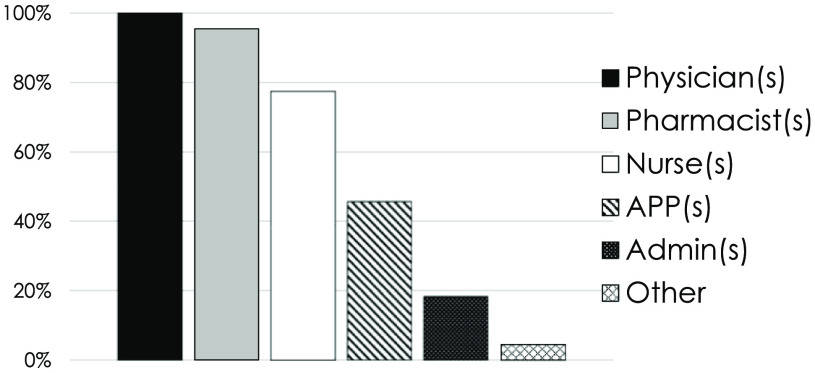
Formal OPAT program composition. Note. OPAT, outpatient parenteral antimicrobial therapy; APP, advanced practice provider.

Of the 27 pharmacists with OPAT practices, 5 stated that there was no formal OPAT team. Of the remaining 22 pharmacists, formal OPAT team composition most frequently included physicians (100%), pharmacists (95.5%), and nurses (77.3%) (Fig. 1). A median of 1 OPAT pharmacist regularly practicing in the OPAT clinic, ranging up to 5 (Table 2). The median pharmacist full-time equivalents (FTEs) dedicated to OPAT was 0.6 (range, 0–2.0). Most (63%) utilized a collaborative practice agreement or held a practice extension (eg, pharmacist clinician licensure) to facilitate independent work. Most OPAT pharmacists (81.5%) shared job functions with other aspects of ID. These included antimicrobial stewardship (81.5%), inpatient ID consultation (59.3%), pharmacokinetic consultations (37.0%), HIV clinic (18.5%), hepatitis B or C clinics (11.1%), and others. Most did not have a dispensing component associated with their practice.

**Table 2.** OPAT Program Demographics

Variable	Result
	(N=27), No.(%)
Pharmacist FTE dedicated to OPAT/COpAT, median (range)	0.6 (0.2–1.0)
Patients enrolled in OPAT/COpAT, median (IQR)	43 (10–65)
**Reporting department**	
Inpatient pharmacy	20 (74.1)
Ambulatory pharmacy	5 (18.5)
College of Pharmacy faculty	3 (11.1)
Infectious diseases	2 (7.4)
Pharmacists with a collaborative practice agreement of other license extension	17 (63.0)
Pharmacists with job sharing with other infectious diseases functions	22 (81.5)
**Responsibility type**	
Antimicrobial stewardship	22 (81.5)
Inpatient infectious diseases consults	16 (59.3)
Pharmacokinetic consults	10 (37.0)
HIV clinic	5 (18.5)
Hepatitis B or C clinic	3 (11.1)
Other outpatient infectious diseases clinic	6 (22.2)
OPAT clinic is a separate clinic from other outpatient infectious diseases clinics	12 (44.4)
Pharmacists with an antimicrobial dispensing component	3 (11.1)
Percentage of OPAT patients, median % (IQR)	75 (50–95)
**Frequency of OPAT patient follow-up**	
Multiple times per week	1 (3.8)
Weekly	5 (19.2)
Every 2 weeks	2 (7.7)
Monthly	11 (42.3)
Other	7 (26.9)
Missing	1
Percentage of COpAT patients, median % (IQR)	10 (1–25)
**Frequency of COpAT patient follow-up**	
Multiple times per week	1 (4.2)
Weekly	2 (8.3)
Every 2 weeks	4 (16.7)
Monthly	8 (33.3)
Other	9 (37.5)
Missing	3
Availability of follow-up as telemedicine	19 (71.4)
**Types of telemedicine**	
Telephone or video conferencing	11 (40.7)
Telephone only	7 (25.9)
Video conferencing only	1 (3.7)

Note. FTE, full-time equivalent; OPAT, outpatient parenteral antimicrobial therapy; COpAT, complex outpatient antimicrobial therapy; IQR, interquartile range.

OPAT programs were robust, with a median of 43 patients (interquartile range [IQR], 10–65) actively enrolled on a given day (Table 2). It was estimated that a median of 75% (IQR, 50%–95%) of patients were on intravenous antimicrobials (OPAT) and median of 10% (IQR, 1%–25%) were on COpAT. Patients on OPAT were usually seen once per month (42.3%) or once weekly (19.2%). Patients on COpAT had more variability, with monthly (33.3%) or nonstandard follow-up intervals (37.5%). Additionally, 70.4% of OPAT programs have access to some form of telemedicine for OPAT follow-ups.

**Fig. 2.**
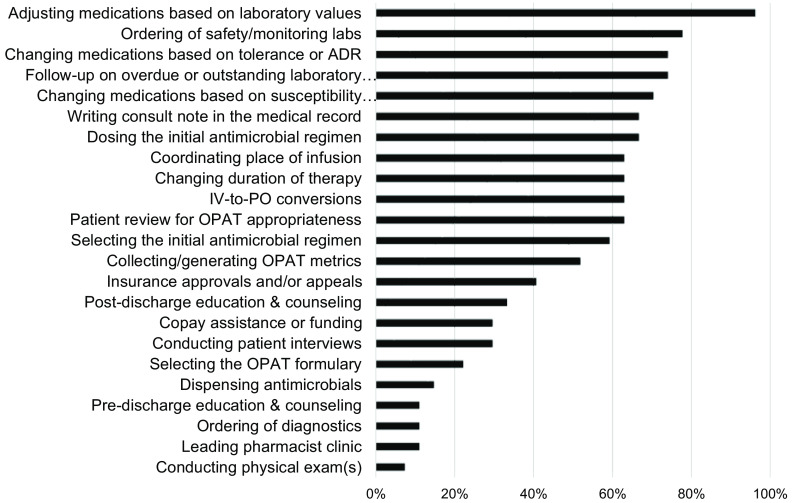
Functions performed by OPAT pharmacists. Note. OPAT, outpatient parenteral antimicrobial therapy; ADR, adverse drug reaction; IV, intravenous; PO, per os (oral). Respondents were asked to indicate which tasks they perform in their OPAT capacity. Respondents could check all that applied.

**Fig. 3.**
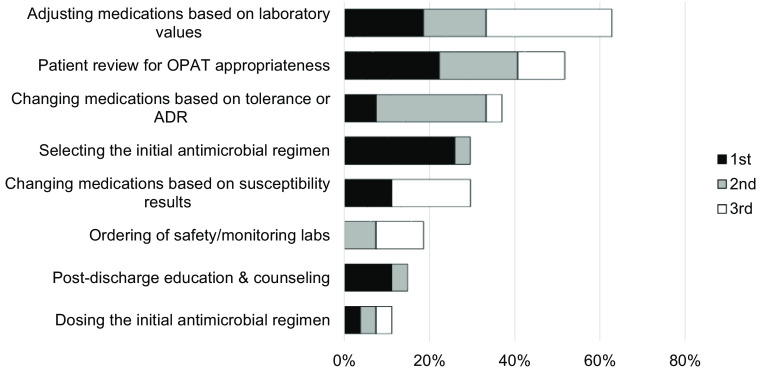
Ranking of importance of OPAT pharmacist functions. Note. OPAT, outpatient parenteral antimicrobial therapy; ADR, adverse drug reactions. Respondents were asked to rank 3 activities as being the most important, second most important, and third most important. Rankings shown here are aggregates of the top 3 choices, combined.

Pharmacists who practiced OPAT were asked to select all functions they performed within their practice (Fig. 2). Adjusting medication dose based on laboratory values was the most frequently cited (96.3%), followed by changing regimen based on patient tolerance or reaction (74.1%) and outreach for outstanding laboratory and safety parameters (74.1%). Additionally, pharmacists were asked to rank various OPAT functions in order of importance. Rankings for first most important, second most important, and third most important were combined for scoring purposes (Fig. 3). Overall, adjusting antimicrobial doses based on safety laboratories was scored as the most important activity OPAT pharmacists performed. Following this activity were patient review for OPAT appropriateness prior to hospital discharge and changing antimicrobial regimens based on tolerance or adverse reaction.

## Discussion

Several national organizations have published OPAT guidelines,^1,6^ including a recent review of antimicrobial stewardship opportunities within OPAT programs.^7^ Furthermore, the IDSA OPAT E-handbook highlights areas that utilize the education and expertise of pharmacists. These include antimicrobial selection, administration, duration of therapy, allergy assessment, laboratory and safety monitoring, pharmacokinetic or pharmacodynamic dosing, antimicrobial stewardship principles, and patient or caregiver counseling.^8^ Recent practice surveys of OPAT physicians indicated that ∼60% of clinics have some sort of pharmacist involvement.^4^ Additionally, one small study demonstrated the inclusion of a pharmacist into an OPAT clinic has been associated with adherence to ID guidelines.^9^

In this survey, OPAT pharmacists performed a wide variety of activities, mostly focusing on clinical aspects. However, what OPAT pharmacists valued as important for OPAT practice did not entirely align with the functions they were performing. OPAT pharmacists were frequently ordering or reaching out for outstanding safety laboratories, but they did not place as high value in this. Although timely laboratory monitoring has been shown to contribute to overall OPAT care,^10^ the unspecialized nature of the task may explain its relatively lower ranking. OPAT pharmacists also placed lesser importance on documentation in the electronic health record. Clear communication is a vital component of successful OPAT programs, and certain aspects of documentation can be automated or templated. Likewise, OPAT pharmacists indicated the high importance of reviewing and selecting the OPAT regimen prior to hospital discharge. However, only ∼60% of OPAT pharmacists were performing these activities. Pharmacists maintain a robust knowledge of antimicrobial properties, including spectrum of activity, pharmacodynamic and alternative dosing strategies, and infusion device modalities. Pharmacists are optimally poised for designing regimens more likely to achieve patient adherence. If able, thoughtful OPAT program redesign should maximize these activities and leverage pharmacists’ strengths.

Interestingly, respondents listed that only 10% of patients were on COpAT. With recent publications showing noninferiority of oral to intravenous regimen one might expect to see more oral regimen utilized, especially if the regimen is being designed by an ID-trained pharmacist and the ongoing coronavirus disease 2019 (COVID-19) pandemic.^11–13^ A likely explanation is that these patients are not followed or followed less frequently by the OPAT team. Notably, some data suggest that an increased rate of adverse drug events with oral regimens that pharmacists are suited to manage.^14,15^ Further research may be warranted to better elucidate this trend.

A new finding from this survey includes the establishment of a benchmark of OPAT pharmacist staffing (FTE) to patient ratio. The survey results reported a median of 0.6 OPAT pharmacist FTE and a median of 43 active patients receiving OPAT. Therefore, we propose a ratio of 1 OPAT pharmacist FTE for every 45–70 patients on OPAT.

Although this is the largest survey of pharmacists practicing in OPAT, there are still many unknowns. Moreover, 27 pharmacists responded that they had OPAT practices. Coupled with programs associated with author institutions, at least 30 pharmacists within the United States have OPAT practices. Also, 118 physicians indicated formal OPAT teams. This could mean that many programs exist without formal pharmacist involvement, indicating an opportunity for growth in the United States, or that the target audience are not members of the ACCP ID PRN, which requires a paid membership. Alternatively, survey participation may have been low due to competing priorities, (eg, COVID-19 response). To facilitate networking and growth, an OPAT pharmacist contact directory has been started, and interested pharmacists can contact the authors for addition. As the inclusion of pharmacists in OPAT services grows, concerted efforts should be made to include pharmacists in multidisciplinary practice surveys.

Expansion of pharmacist involvement in OPAT may be mutually beneficial in advancement of pharmacists’ practice and work shifting or sharing with professional colleagues (physicians, advanced practice providers). Although collaborative practice agreements or license extension agreements may facilitate OPAT work, only 63% of those working in OPAT reported using either of those in their practice. Pharmacist OPAT participation may occur within the usual allowances of pharmacists’ licensure.

Pharmacist involvement in OPAT is an emerging trend within many different types of OPAT programs. Tasks performed by OPAT pharmacists varied significantly, and most commonly, clinical (nondispensing) functions are being performed. We propose a ratio of 1 OPAT pharmacist FTE for every 45–70 patients receiving OPAT. Opportunity exists for further expansion of pharmacists in OPAT programs.

## Supplementary material

For supplementary material accompanying this paper visit https://doi.org/10.1017/ash.2022.40. click here to view supplementary material
