# Supplementary material for: Survey of pharmacists on their roles and perceptions of outpatient parenteral antimicrobial therapy in the United States
Source: Antimicrob Steward Healthc Epidemiol. 2022 Apr 22;2(1):e69. doi: 10.1017/ash.2022.40 (PMC9726508; doi:10.1017/ash.2022.40)
Supplement: Supplementary file 1 [file S2732494X22000407sup001.pdf]

Pharmacists Practice in OPAT/COpAT

We are conducting research on the involvment of pharmacists in Outpatient Parenteral Antimicrobial Therapy (OPAT) and Complex Antimicrobial Outpatient Therapy (COpAT). This will help us understand the current level of pharmacists involvement in OPAT/COpAT in the United States. The full survey should only take about 10 minutes.

Survey participants will be offered an opportunity to enter a raffle for one of five $50 Visa cash cards. You are not required to complete all questions and can skip questions. Those whom have no direct involvement in OPAT/COpAT may also participate and enter in the cash card raffle.

You may only take the survey once, but you can edit your responses until the survey is closed on March 31, 2021 midnight CDT.

If you have any questions about the survey, please email us: kryan2@salud.unm.edu or rivera.christina@mayo.edu

---

Do you currently practice in the United States?

- ○ Yes
- ○ No

Do you actively participate in an Outpatient Parenteral Antimicrobial Therapy clinic (OPAT)?

- ○ Yes
- ○ No

Domain 1: RPh Background & OPAT Role

Please select your pharmacist degree(s): (choose all that apply)

- ☐ Bachelor of Pharmacy
- ☐ Master of Pharmacy
- ☐ Doctor of Pharmacy
- ☐ Other, enter free text

If other, enter your pharmacy degree:

____

Please select any post-graduate training: (choose all that apply)

- ☐ PGY1
- ☐ PGY2-Infectious Disease
- ☐ PGY2-Ambulatory Care
- ☐ PGY2 Other
- ☐ ID Fellowship
- ☐ Antimicrobial Stewardship Certificate
- ☐ None

How many years have you been a licensed pharmacist?

- ○ < 5 years
- ○ 5-10 years
- ○ 10-15 years
- ○ >15 years

How many years have you been practicing in Infectious Diseases (ID)?

- ○ < 5 years
- ○ 5-10 years
- ○ 10-15 years
- ○ >15 years

How many years have you been practicing in OPAT/COpAT?

- ○ < 5 years
- ○ 5-10 years
- ○ 10-15 years
- ○ >15 years

In what state do you practice pharmacy?

◯ Alabama
◯ Alaska
◯ American Samoa
◯ Arizona
◯ Arkansas
◯ California
◯ Colorado
◯ Connecticut
◯ Delaware
◯ Florida
◯ Guam
◯ Georgia
◯ Hawaii
◯ Idaho
◯ Illinois
◯ Indiana
◯ Iowa
◯ Kansas
◯ Kentucky
◯ Louisiana
◯ Maine
◯ Maryland
◯ Massachusetts
◯ Michigan
◯ Minnesota
◯ Mississippi
◯ Missouri
◯ Montana
◯ Nebraska
◯ Nevada
◯ New Hampshire
◯ New Jersey
◯ New Mexico
◯ New York
◯ North Carolina
◯ North Dakota
◯ Northern Marian Islands
◯ Ohio
◯ Oklahoma
◯ Oregon
◯ Pennsylvania
◯ Puerto Rico
◯ Rhode Island
◯ South Carolina
◯ South Dakota
◯ Tennessee
◯ Texas
◯ Utah
◯ U.S virgin Islands
◯ Vermont
◯ Virginia
◯ Washington
◯ Washington DC
◯ West Virginia
◯ Wisconsin
◯ Wyoming

How many pharmacist(s) regularly practice in the OPAT/COpAT clinic?

____

How many pharmacist FTEs (full time equivalents) are dedicated to OPAT/COpAT?

____

Does the OPAT/COpAT pharmacist have a collaborative practice agreement or other license extensions?	◯ Yes<br>◯ No
Does the OPAT/COpAT pharmacist have job sharing with other ID functions?	◯ Yes<br>◯ No
Please select all other functions that apply:	☐ Antimicrobial stewardship<br>☐ HIV clinic<br>☐ Hepatitis B or C clinic<br>☐ Other outpatient ID clinic<br>☐ Inpatient ID consults<br>☐ Pharmacokinetic consults
What best describes your OPAT/COpAT practice setting:	◯ Academic medical center/hospital<br>◯ Community hospital<br>◯ Veteran's Affairs/Government hospital<br>◯ Infusion clinic - hospital owned<br>◯ Infusion clinic - private owned/for profit<br>◯ Other: free text
What best describes your current practice setting:	◯ Academic medical center/hospital<br>◯ Community hospital<br>◯ Veteran's Affairs/Government hospital<br>◯ Infusion clinic - hospital owned<br>◯ Infusion clinic - private owned/for profit<br>◯ Other: free text
If Other please describe your practice setting:	__________________________________
Which department do you report to? (select all that apply)	☐ Pharmacy - Inpatient<br>☐ Pharmacy - Ambulatory<br>☐ Pharmacy - Specialty<br>☐ Pharmacy - Other<br>☐ College of Pharmacy Faculty<br>☐ Infectious Diseases<br>☐ Other: free text
If Other please list to whom you report:	__________________________________

Domain 2: Team related

Please select all members of your institutional OPAT/COpAT team:	☐ Pharmacist(s)<br>☐ Nurse(s)<br>☐ Physician(s)<br>☐ Advanced practice providers: nurse practitioner, physician's assistant<br>☐ Administrative assistant<br>☐ Other: free text<br>☐ No formal OPAT team<br>☐ OPAT team exists, but I am unsure of members
if other please list additional OPAT/COpAT team members:	__________________________________

Does the OPAT/COpAT program involve an antibiotic dispensing component? (e.g. does OPAT pharmacist prepare medications, or verify medications prior to patient administration?)	◯ Yes<br>◯ No
For clinical issues that arise outside of scheduled clinic times who is responsible for addressing these issues?	☐ Ordering provider<br>☐ On call OPAT specific provider (pharmacist vs APP)<br>☐ On call ID attending (e.g. inpatient team)<br>☐ No on call services

Domain 3: Clinic Functions

On a typical day, about how many patients are actively enrolled in OPAT/COpAT?	___________________________________
Is the OPAT clinic separate from other ID clinics (i.e HIV, Hepatitis C, Non-Tuberculosis Mycobacterium)?	◯ Yes<br>◯ No
In thinking of only your OPAT and COpAT patients, please indicate the % of OPAT patients in your practice:	0 50 100<br>*(Place a mark on the scale above)*
In thinking of only your OPAT and COpAT patients, please indicate the % of COpAT patients in your practice:	0 50 100<br>*(Place a mark on the scale above)*
How frequently are patients on OPAT seen in follow-up?	◯ Multiple times a week<br>◯ Weekly ◯ Every 2 weeks<br>◯ Monthly ◯ Other
If other please list the frequency of patient follow-up:	___________________________________
How frequently are patients on COpAT seen in follow-up?	◯ Multiple times a week<br>◯ Weekly ◯ Every 2 weeks<br>◯ Monthly ◯ Other
If other please list the frequency of patient follow-up:	___________________________________
Does your OPAT/COpAT team follow-up via scheduled telemedicine (select all that apply)?	◯ No<br>◯ Yes via telephone only<br>◯ Yes via video conferencing only<br>◯ Yes via telephone or video conferencing

Please check all functions you perform as the OPAT pharmacist: (including recommendations to prescribing clinicians and independent practice)	☐ Patient review for OPAT appropriateness prior to discharge.<br>☐ Predischarge education and counseling<br>☐ Transition of Care/ Post discharge education and counseling<br>☐ Dispensing of antimicrobials<br>☐ Patient interviews<br>☐ Conducting physical exams<br>☐ Selecting the initial antibiotic regimen<br>☐ Dosing the initial antibiotic regimen<br>☐ Ordering of safety/monitoring labs<br>☐ Ordering of diagnostics (radiology, etc)<br>☐ Adjusting medication doses based on lab values<br>☐ Changing medications based on susceptibility results<br>☐ Changing medications based on patient tolerance or reaction<br>☐ Insurance approvals and/or appeals<br>☐ Co-pay assistance or funding<br>☐ IV to PO conversions<br>☐ Changing duration of therapy<br>☐ Coordinating place of infusion (e.g., coordinating outpatient dalbavancin or oritavancin infusion)<br>☐ Lead a pharmacist clinic (patient has appointment with pharmacist)<br>☐ Write consult notes in the medical record<br>☐ Follow-up on overdue or outstanding laboratory values<br>☐ Select OPAT drug formulary<br>☐ Collect/generate OPAT metrics

Please select what you feel is the MOST IMPORTANT function of an OPAT Pharmacist:

- ◯ Patient review for OPAT appropriateness prior to discharge.
- ◯ Predischarge education and counseling
- ◯ Transition of Care/ Post discharge education and counseling
- ◯ Dispensing of antimicrobials
- ◯ Patient interviews
- ◯ Conducting physical exams
- ◯ Selecting the initial antibiotic regimen
- ◯ Dosing the initial antibiotic regimen
- ◯ Ordering of safety/monitoring labs
- ◯ Ordering of diagnostics (radiology, etc)
- ◯ Adjusting medication doses based on lab values
- ◯ Changing medications based on susceptibility results
- ◯ Changing medications based on patient tolerance or reaction
- ◯ Insurance approvals and/or appeals
- ◯ Co-pay assistance or funding
- ◯ IV to PO conversions
- ◯ Changing duration of therapy
- ◯ Coordinating place of infusion (e.g., coordinating outpatient dalbavancin or oritavancin infusion)
- ◯ Lead a pharmacist clinic (patient has appointment with pharmacist)
- ◯ Write consult notes in the medical record
- ◯ Follow-up on overdue or outstanding laboratory values
- ◯ Select OPAT drug formulary
- ◯ Collect/generate OPAT metrics

Please select what you feel is the SECOND MOST important function of an OPAT Pharmacist:

- ◯ Patient review for OPAT appropriateness prior to discharge.
- ◯ Predischarge education and counseling
- ◯ Transition of Care/ Post discharge education and counseling
- ◯ Dispensing of antimicrobials
- ◯ Patient interviews
- ◯ Conducting physical exams
- ◯ Selecting the initial antibiotic regimen
- ◯ Dosing the initial antibiotic regimen
- ◯ Ordering of safety/monitoring labs
- ◯ Ordering of diagnostics (radiology, etc)
- ◯ Adjusting medication doses based on lab values
- ◯ Changing medications based on susceptibility results
- ◯ Changing medications based on patient tolerance or reaction
- ◯ Insurance approvals and/or appeals
- ◯ Co-pay assistance or funding
- ◯ IV to PO conversions
- ◯ Changing duration of therapy
- ◯ Coordinating place of infusion (e.g., coordinating outpatient dalbavancin or oritavancin infusion)
- ◯ Lead a pharmacist clinic (patient has appointment with pharmacist)
- ◯ Write consult notes in the medical record
- ◯ Follow-up on overdue or outstanding laboratory values
- ◯ Select OPAT drug formulary
- ◯ Collect/generate OPAT metrics

Please select what you feel is the THIRD MOST important function of an OPAT Pharmacist:

◯ Patient review for OPAT appropriateness prior to discharge.
◯ Predischarge education and counseling
◯ Transition of Care/ Post discharge education and counseling
◯ Dispensing of antimicrobials
◯ Patient interviews
◯ Conducting physical exams
◯ Selecting the initial antibiotic regimen
◯ Dosing the initial antibiotic regimen
◯ Ordering of safety/monitoring labs
◯ Ordering of diagnostics (radiology, etc)
◯ Adjusting medication doses based on lab values
◯ Changing medications based on susceptibility results
◯ Changing medications based on patient tolerance or reaction
◯ Insurance approvals and/or appeals
◯ Co-pay assistance or funding
◯ IV to PO conversions
◯ Changing duration of therapy
◯ Coordinating place of infusion (e.g., coordinating outpatient dalbavancin or oritavancin infusion)
◯ Lead a pharmacist clinic (patient has appointment with pharmacist)
◯ Write consult notes in the medical record
◯ Follow-up on overdue or outstanding laboratory values
◯ Select OPAT drug formulary
◯ Collect/generate OPAT metrics

Respondent demographics

What is your gender?

◯ Female
◯ Male
◯ Transgender female
◯ Transgender male
◯ Non-binary
◯ Gender fluid
◯ Prefer not to answer

What is your race? (Mark all that apply)

☐ American Indian or Alaska Native
☐ Asian
☐ Black or African American
☐ Native Hawaiian or Other Pacific Islander
☐ White
☐ Prefer not to answer

What is your ethnicity?

◯ Hispanic or Latino
◯ Not Hispanic or Latino
◯ Prefer not to answer

Would you like to be entered in the drawing for a $50 Visa cash card?

◯ Yes
◯ No

Please enter your preferred email for the Visa cash card lottery: (your email will ONLY be used for the raffle and NOT shared outside the study team)

____
